# An Energy Efficient and Secure IoT-Based WSN Framework: An Application to Smart Agriculture

**DOI:** 10.3390/s20072081

**Published:** 2020-04-07

**Authors:** Khalid Haseeb, Ikram Ud Din, Ahmad Almogren, Naveed Islam

**Affiliations:** 1 Department of Computer Science, Islamia College Peshawar, Peshawar 25000, Pakistan; khalid.haseeb@icp.edu.pk (K.H.); naveed.islam@icp.edu.pk (N.I.); 2 Department of Information Technology, The University of Haripur, Haripur 22620, Pakistan; ikramuddin205@yahoo.com; 3 Chair of Cyber Security, Department of Computer Science, College of Computer and Information Sciences, King Saud University, Riyadh 11633, Saudi Arabia

**Keywords:** smart agriculture, cluster heads, energy efficiency, data security, signal strength

## Abstract

Wireless sensor networks (WSNs) have demonstrated research and developmental interests in numerous fields, like communication, agriculture, industry, smart health, monitoring, and surveillance. In the area of agriculture production, IoT-based WSN has been used to observe the yields condition and automate agriculture precision using various sensors. These sensors are deployed in the agricultural environment to improve production yields through intelligent farming decisions and obtain information regarding crops, plants, temperature measurement, humidity, and irrigation systems. However, sensors have limited resources concerning processing, energy, transmitting, and memory capabilities that can negatively impact agriculture production. Besides efficiency, the protection and security of these IoT-based agricultural sensors are also important from malicious adversaries. In this article, we proposed an IoT-based WSN framework as an application to smart agriculture comprising different design levels. Firstly, agricultural sensors capture relevant data and determine a set of cluster heads based on multi-criteria decision function. Additionally, the strength of the signals on the transmission links is measured while using signal to noise ratio (SNR) to achieve consistent and efficient data transmissions. Secondly, security is provided for data transmission from agricultural sensors towards base stations (BS) while using the recurrence of the linear congruential generator. The simulated results proved that the proposed framework significantly enhanced the communication performance as an average of 13.5% in the network throughput, 38.5% in the packets drop ratio, 13.5% in the network latency, 16% in the energy consumption, and 26% in the routing overheads for smart agriculture, as compared to other solutions.

## 1. Introduction

In various domains, the technology of wireless sensor network (WSN) [1,2,3] has been used in an efficient way to improve network performances. The main reason to uses different sensors in the environmental field due to their manageable and easy configuration setup [4,5,6,7]. Additionally, the sensor nodes operate autonomously and construct the network infrastructure in an ad-hoc manner. In such infrastructure, nodes have not a stable network topology and they can join the more suitable neighbor for data transmission based on some factors. The sensor nodes sense the observing data and forward towards BS with the help of some gateway and cluster heads. These cluster heads have a role of aggregating the received data packets and relay towards BS. The cluster heads effectively construct a single-hop or multi-hop path to BS and work as a focal point in entire data transmission. Furthermore, the cluster heads store the received data in its memory and follow the store and forward mechanism. The end-users access the centralized BS via the Internet or different web-based applications to retrieve the required observing data [8,9,10,11].

**Figure 1:**
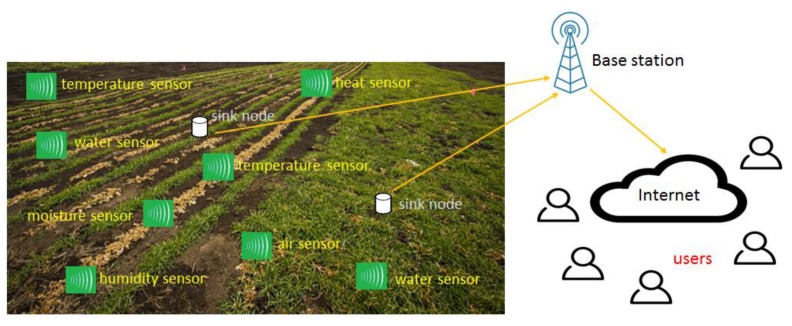
Smart agricultural environment based on wireless sensor network (WSN).

During data transmission, the deployed sensors can be static or mobile. The static sensors are also referred to as non-adaptive and their constructed routing tables are fixed. While on the other hand, the routing tables of mobile sensors are dynamic and frequently updated when any change incurs in the network topology. The static routing solutions are more secure when compared to dynamic routing; however, the solutions that are based on the static algorithms are not appropriate for large regions and network scalability [12,13]. In recent years, the technology of IoT has been merged a lot with other fields to improved communication in terms of network throughput, resource utilization, and load distribution [14,15,16]. In IoT, many physical objects are attached to convert the information while using the Internet. Moreover, the technology of WSN provides the foundation for IoT systems and supports in observing and forwarding the conditions for the physical environment [17,18,19]. Figure 1 illustrates the scenario for smart agriculture based on various sensors, sink nodes, BS, Internet, and users.

The drastic changes in climate negatively impact the agriculture eco-system causing heaving rains, droughts, floods, and abrupt weather conditions [20,21]. These changes are deriving threats to agriculture-related food security in the developing as well as the developed world. The climates related challenges that are faced by agriculture can be overcome by adopting smart agriculture using IoT devices, which can increase agriculture yields and productions. The use of sensors in agriculture has been introduced in the last few decades as an offline data collector [22,23,24]. The offline sensor infrastructure collects data and provides sufficient information for making good decisions to overcome future yields or for the next year crops, yet they are unable to provide data regarding frequent changes in the environment, threatening the agriculture yields.

This article is aimed at using state of the art IoT based sensor infrastructure to collect data from the environment and securely transfer the data to the BS for efficient decisions. In the proposed framework, wireless agriculture sensors are scattered in the agriculture land for extracting different information related to soil composition, like humidity, temperature, moisture levels, and water level finders. This information is securely transmitted to the cluster heads, which works as memory buffers or storages to forward data towards BS. Upon the reception of data by the BS securely, the BS can provide up to date information to users for an efficient decision with minimum time. The proposed framework offers an energy-efficient and reliable routing to automate agriculture productions with minimum farmer’s burden. The observing data of agricultural sensors are routed towards BS intelligently and securely, which improves the monitoring and productivity of the agricultural land. The simulated experiments for the proposed framework demonstrated outperformed results when compared to existing solutions that are based on different network parameters.

This article is outlined in multiple subsections: Section 2 provides the background work and problem statement, Section 3 explains the proposed framework, Section 4 shows the simulation setup and detailed of parameters, Section 5 provides the experimental results along with discussion, and Section 6 provides the conclusion and the future work in this domain.

## 2. Background

In the recent era, the technologies of WSN have been applied by different fields because of their low cost, easy deployment, and cost-effective environment [25,26]. In WSN, a large number of sensor nodes are scattered in observing the field to sense the needed data. All of the data are gathered and forwarded towards BS via single or multi-hop adopted data transmission paradigm for post-analysis. Today, the field of agriculture performs a vital role in the improvement and economic growth of any country. Therefore, the field of agriculture should be exploited with some modern technologies, such as IoT-based WSN to reduce time and human efforts, and increase the agricultural throughput in the quality manner [27]. In agriculture land, different kinds of sensors are used for determining the soil, weather, wetness, and temperature conditions. Although, the field of WSN has been exploited by many researchers in the domain of agriculture to improve its performance and reduce the farmer’s burden [28,29]. However, the deployed sensors have limited constraints in terms of memory, processing, transmitting and energy power. Besides, data protection is another major research challenge for WSN based applications because of their undependable, uncontrolled, and free-space communication foundation.

In WSN [30,31,32], many researchers have proposed different clustering schemes that aimed to prolong network lifetime and efficient data transmission [33,34,35]. In such schemes, WSN divided into various regions and each region has one cluster head, which aims to gather and forward the sensory information towards BS. Furthermore, most of the sensor nodes moved to sleep mode for prolonging network lifetime. Low energy adaptive cluster hierarchy (LEACH) was proposed by [36], aiming to introduce the concept of a cluster-based approach and improve energy efficiency as compared to traditional approaches. The role of the cluster head is randomly rotated and, accordingly, the LEACH protocol balances the energy consumption among the sensor nodes. In [37], the authors proposed the analytic hierarchy process (AHP), which aims to centralize the process of cluster head selection mechanism. Residual energy, mobility, and distance towards cluster centroid are considered to be the main factors for the selection process of cluster heads. The proposed solution significantly improved network lifetime in the comparison of other solutions. The authors in [38] proposed an energy-efficient k-means technique (EKMT) and determine the optimal cluster heads. The selected cluster heads are closer to the cluster’s member as well as the BS. The proposed solution offers to decrease the communication distance among nodes and improve the network lifetime. However, the proposed solution is not efficient in an insecure and unrestricted space environment, as it is open to malicious extortions and might be harmful to sensors data. 

Authors [39] proposed cluster head selection in wireless sensor networks while using a fuzzy environment. The proposed solution based on the fuzzy multiple attribute decision making (MADM) approach and uses residual energy, distance to BS, and the number of neighbor’s factors for selecting the cluster heads. A simulated network lifetime is prolonged than hierarchical agglomerative clustering (DHAC) [40] protocol under a homogeneous background. An improved chain-based clustering hierarchical routing (ICCHR) algorithm that is based on LEACH [41] consists of cluster formation, cluster head selection, chain formation, and data transmission phases. The selection and distribution of cluster heads in the proposed protocol are non-optimal and they cause extra energy consumption. Based on simulation results, the proposed ICCHR algorithm decreases the ratio of energy consumption, evenly distributing the load among sensor nodes. The authors in [42] proposed an optimized zone-based energy-efficient routing protocol for mobile sensor networks (OZEEP). The main aim of the proposed solution is to improve the network performance in terms of cluster formation and cluster head selection based on distance, density, mobility, and energy factors. The proposed solution achieves balanced energy consumption and improved network lifetime; however, the evaluation of wireless links is missing in the proposed solution. Moreover, security measurements are also not taken into consideration, which results in frequent links and routing failures.

The authors [43] proposed the particle swarm optimization-energy efficient-based cluster head selection (PSO-ECHS) protocol, which prolongs network lifetime and network stability. In the proposed PSO-ECHS protocol, the cluster heads are selected using fitness function, which comprises residual energy, distance from sensor nodes to neighbors, and distance from sensor nodes to BS. The cluster heads are selected based on minimum fitness value and, afterward, the cluster formation phase is initiated. All of the selected cluster heads announced their advertisement message and normal nodes joined them to formulate the clusters. In [44], the authors proposed an energy-efficient centroid based routing protocol (EECRP) to achieve data transmission while using wireless sensor networks. In the proposed protocol, BS initiates the process to compute the centroid position and split the network field into various clusters. In the beginning, the node that is closer to the centroid position is selected as the initial cluster head and the role of the cluster head is shifted by determining the new centroid position. Besides, the proposed protocol also decreases the energy consumption for long-distance data communication. In [45], the authors proposed a secure user authentication and key agreement scheme while using WSN, which aims to securely monitor the agriculture domain. The proposed protocol is validated using Burrows-Abadi-Needham (BAN) logic and the simulation results demonstrate better improvement in terms of security against malicious attacks. However, in most of the other existing works, the proposed solution also does not consider the performance of links in data transmission and leads to packets drop and latency ratio.

It is seen from the discussed studies that sensor nodes are very limited in terms of battery power, transmission, storage, and processing resources. Additionally, it is not possible to replace the components of the sensor nodes in dense and large regions. These low powered sensor nodes have significant impact on the performance of the network in terms of delivery ratio and energy consumption. Furthermore, most of the WSN based smart agriculture applications need the required information in an efficient and timely manner. Therefore, optimizing network lifetime with stable routing and data delivery performance are most of the important research challenges in WSN based applications. Additionally, the selection of cluster heads performs a very important role to balance the load distribution and energy consumption in the WSN based agricultural land. The cluster heads that are one hop away from BS exhaust their energy resource much quickly and lead to the energy hole issue, which degrades network throughput and increases the latency ratio. Moreover, it is observed from the existing work that most of the solution does not consider the strength of the signal in data routing, which significantly increases the packet drop ratio and instability in the network region. Accordingly, most of the energy power of sensor nodes is dissipated due to the selection of poor wireless carrier channels and it leads to frequent retransmissions of agricultural sensors’ data. Furthermore, end users obtained the information from the IoT based WSN while using the Internet, which is full of malicious actions and network information can be disclosure to anonymous nodes. Therefore, data security is another important research aim for a WSN based smart agriculture to achieve network reliability and trustworthiness. Consequently, an energy-efficient and secure IoT based WSN framework must be layout and developed for smart agriculture to optimize network lifetime with reliable delivery performance to end-users. Besides, a trustworthy mechanism can increase the security of individual sensors and formulate realistic end-to-end routing paths. Therefore, the combined concept of energy efficiency with lightweight data security among the low power sensor nodes can improve the efficacy of the IoT-based WSN framework for smart agriculture. 

## 3. Proposed Energy Efficient and Secure IoT-based WSN Framework for Smart Agriculture

Several researchers have used the technology of WSN in various domains to sense the environmental data. WSN has also played a vital role in the observation and management of agricultural land in terms of crops, climate, water usage, etc. However, the agricultural land still has some challenges, such as energy efficiency, data routing, and security, due to the limited battery power of sensors and open transmission medium. The basic aim of the proposed solution to develop an energy-efficient and secure IoT based WSN framework for the monitoring and production of agricultural land. In the proposed framework, an appropriate clusters heads are selected based on the optimal decision function. Furthermore, the SNR factor also incorporated in the decision to compute the strength of wireless signals and increase the successful ratio of sensors packets. Our proposed framework offers reliable and energy-efficient methods for the improvement of large-sized agricultural land. Moreover, the proposed framework also accumulated the data security among agricultural sensors to cluster heads and from cluster heads to BS based on secret keys while using the linear congruential generator [46,47], which requires minimal memory and processing time. Accordingly, our proposed framework ensures the trade-off among energy consumption, reliable and secure routing for the agricultural filed.

**Figure 2:**
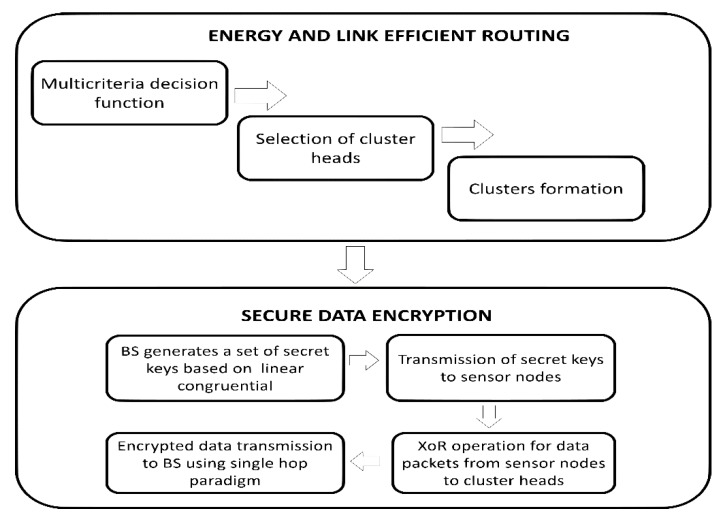
The research design of proposed energy-efficient and secure IoT-based WSN framework for smart agriculture.

The research design of the proposed framework is explained in this section. The proposed framework comprises of two main components. In the first component, the agricultural sensors are dispersed to gather information. The sensor nodes are heterogeneous in terms of residual energy, such that the energy level of the heterogeneous nodes is higher than normal sensor nodes. The agricultural sensors are dispersed into various distant areas and each area consists of one cluster head. The role of the cluster head is to receive the information from agriculture land and forward towards BS in a fault-tolerant and energy-efficient manner. The proposed framework balances the load among agricultural sensors and it selects suitable cluster heads based on multi-criteria decision function. Moreover, our proposed framework makes use of a single-hop transmission instead of a multi-hop paradigm to reduce the network bottlenecks and network latency. In the second component, the proposed security mechanism exploits symmetric data encryption between agricultural sensors and presents a robust transmission in the field using pseudorandom number generation. Figure 2 demonstrates the research design of the proposed framework for smart agriculture.

### 3.1. Network Assumptions

Before discussing the proposed Energy Efficient and Secure IoT Based WSN Framework for Smart Agriculture, some network assumptions are highlighted, as follows:

- N number of agriculture sensors are dispersed in the observing squared sized area.
- All the agriculture sensors and BS remain fixed after the nodes deployment.
- Transmission links are symmetric.
- Agriculture sensors are heterogeneous in terms of energy resources.
- BS has the most powerful node with unlimited resources.
- The location of agriculture sensors is determined using Global Positioning System (GPS).

### 3.2. Energy and Link Efficient Routing

This section presents discussion on the design of the proposed energy and link efficient routing, which includes two main levels. The first level is based on a multi-criteria decision function for the selection of optimal cluster heads. Afterwards, the least energy consumption nodes are formulated into different clusters. The second level of the proposed energy and link efficient routing is to make the routing channel more stable for a longer time, which prevents the wireless link from misbehaving activities from cluster heads to the BS. Our proposed framework uses the node energy$\;e_{i}$, Signal to Noise Ratio $SNR_{i,BS}$, and distance to BS $d_{i,BS}$ as a multi-criteria decision-making function f(n) for the selection of cluster heads, as given in equation 1. The aim of using SNR [48,49] is the proposed framework is to determine the strength of the signal and efficiently increase the delivery performance. SNR is defined as the ratio of the Received Signal Strength Indicator (RSSI) to background noise. Let us consider that $RSSI_{i}$ is the received signal strength indicator and $Bn_{i}$ denotes the background noise for link i accordingly, the value of $SNR_{i}$ can be computed by $RSSI_{i}/Bn_{i}$. The link with the least value of $SNR_{i}$ is chosen as being more appropriate for data transmission.

(1) $$f\left(n\right)=e_{i}+(1/d_{i,BS})+(1/SNR_{i})$$

The value of f(n) is normalized in the range of [0…1] using 1−f(n).

In the proposed framework, BS maintains a global table that comprises information regarding agricultural sensors. The store information consists of node position, distance from BS, residual energy and SNR factors. Based on the stored information, BS evaluates f(n) and generates a normalized score. Afterward, BS chooses the highest scored sensor node in agriculture land as an initial cluster head. Accordingly, BS maps the entries against the nodes as selected cluster heads based on f(n). The rest of the nodes that are not marked as cluster heads are considered to be normal nodes. Subsequently, BS formulate the one-hop nodes from each cluster head as its member and accordingly updates the global table. In the proposed framework, all the agriculture sensors gathered the required data and forwarded it to their cluster heads. Afterward, cluster heads exploit a single-hop mechanism to transmit the data towards BS, which further interconnects to the Internet and the end-users can obtain the data regarding agricultural land for further analysis.

### 3.3. Secure Data Transmission from Agriculture Sensors towards BS

In the proposed framework, data from the agriculture sensors are routed while using a secure and trusted network towards the cluster heads and further towards the BS. In this framework, the BS generates secret keys using the recurrence of the linear congruential equation that is given by Equation (2).

(2) $$Y_{n+1}=\left(\alpha Y_{n}+\beta \right)\;mod\;m$$

where $Y_{i}$ are the generated secret random values for sensor node $n_{i}$, m is the modulus parameter which must be greater than 0, α is the multiplier parameter and it must be greater than zero and less than the modulus m, β is the increment parameter and it must be greater or equal to zero and less than the modulus m, and $Y_{0}$ is the seed value and it must also be greater or equal to zero and less than the modulus m. Accordingly, all of the sensor nodes are provided secret keys using equation 2. Afterward, when the sensor node $n_{i}$ send data $m_{i}$ to the cluster head CHj, it is encrypted while using Equation (3).

(3) $$E_{j}\left(m_{i}\right)=m_{i}⊕Y_{i}$$

where ⊕ is the XOR operation between the data $m_{i}$ from the agriculture sensor node $n_{i}$ towards CHj. The encrypted data $E_{j}\left(m_{i}\right)$ is further transmitted towards the BS, which can decrypt it by taking the XOR with the key $Y_{i}$ as given in equation 4.

(4) $$D_{j}\left(m_{i}\right)=E_{j}\left(m_{i}\right)⊕Y_{i}$$

## 4. Simulation Setup with Experimental Results

This section presents the default simulation parameters [50,51,52] that are used in the experimental analysis of the proposed framework against relevant solutions i.e., PSO-ECHS [43] and EECRP [44]. The simulation experiments are executed using Network Simulator2 (NS2) [53,54], which is a well-known open-source and best simulation tool to analyze network routing and communication. Table 1 illustrates the simulation parameters along with their default values. The simulation results are evaluated based on a varying number of rounds. The period of a single simulation round is set to 20 s. Additionally, the number of agriculture sensors and anonymous nodes is set to 100 and 15, respectively. All of the agricultural sensors, i.e., temperature sensors, light sensors, soil moisture sensors, location sensors, airflow sensors, etc., and anonymous nodes are scattered randomly. The number of agricultural sensors is set to 100. The number of malicious nodes is fixed to 15. Packet size (k) and payload size are set to 64 bits and 256 bytes. The residual energy of agriculture sensors is non-uniform, i.e., 2j to 4j. The data traffic between the sensor nodes is a type of Constant Bit Rate (CBR) and a transmission range of agriculture sensors is fixed to 20 m.

## 5. Experimental Results and Discussion

The section explains the experimental results between the proposed framework and the existing solution. The performance of the proposed framework with other solutions is measured based on network throughput, packets drop ratio, network latency, energy consumption, and routing overheads.

### 5.1. Analysis of Network Throughput with Discussion

**Figure 3:**
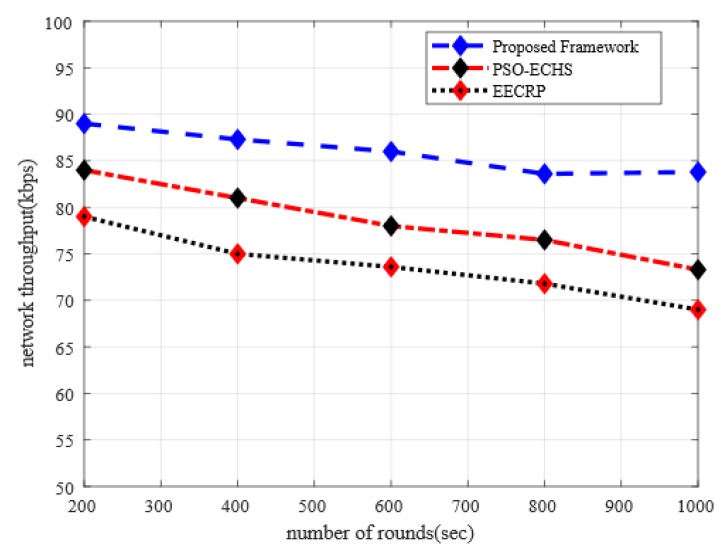
The impact of the simulation rounds on network throughput.

In Figure 3, the proposed framework is compared with an existing solution in terms of the network throughput under varying simulation rounds. It is seen from the simulation experiments that the proposed framework improved the performance of network throughput in comparison with the existing solutions by 10% and 17%, respectively. The improved performance of network throughput is due to the robust and link-aware cluster heads. Unlike other solutions that do not consider the measurement of signals strength between sensor nodes, the proposed framework incorporates SRN factor in an intelligent selection decision for choosing cluster heads, which increases the ratio of packet delivery in agricultural land due to their robust behavior. Moreover, the increase in network throughput for the proposed framework against existing solutions is due to the sharing of data encryption secret keys that are based on the linear congruential generator, such that proposed solution leads to efficient network throughput with consistent network connectivity. Additionally, the proposed framework measures the SNR ratio among nodes for a particular data transmission link, which considerably affects the transmission of agricultural data towards BS.

### 5.2. Analysis of Packets Drop Ratio with Discussion

**Figure 4:**
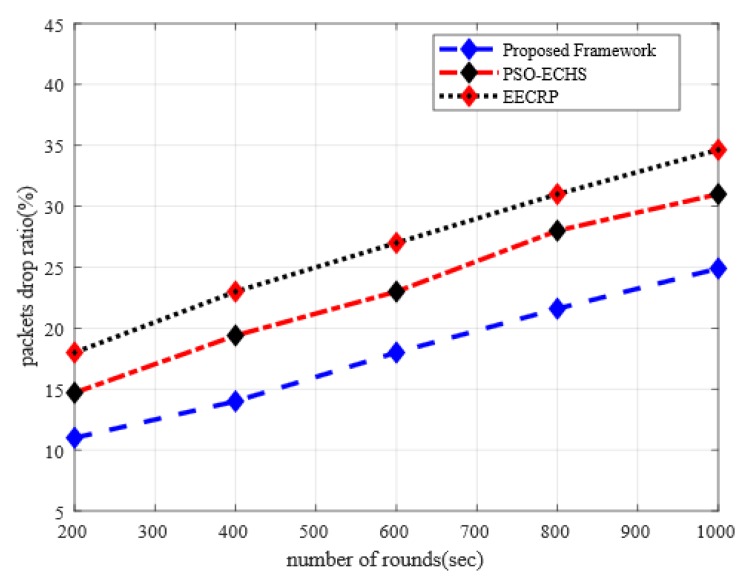
The impact of simulation rounds on the packets drop ratio.

Figure 4 demonstrates the simulation results of the proposed framework with other solutions in terms of the packet drop ratio. It is observed from the results that the proposed framework improved the packet drop ratio by 29% and 48% when compared to the existing solutions. The existing solution overlooked the importance of transmission link factor thereby results in increasing drop rates. Our proposed framework makes use of the measurement of signal strength along with the residual energy of sensor nodes during data forwarding, which results in offering efficient routing performance. Furthermore, the proposed framework improves the ratio of packets delivery by evaluating the multi-criteria decision function and eliminating the congested nodes for data routing. Additionally, the proposed framework incorporates the distance to the BS factor and reduces the length of routing paths and achieves a better packet reception ratio. Besides, the proposed framework makes the data transmission more secure with limited computational overheads on the part of sensor nodes, which decreases the chances of anonymous nodes to drop the data packets and increases the performance of data privacy.

### 5.3. Analysis of Network Latency with Discussion

**Figure 5:**
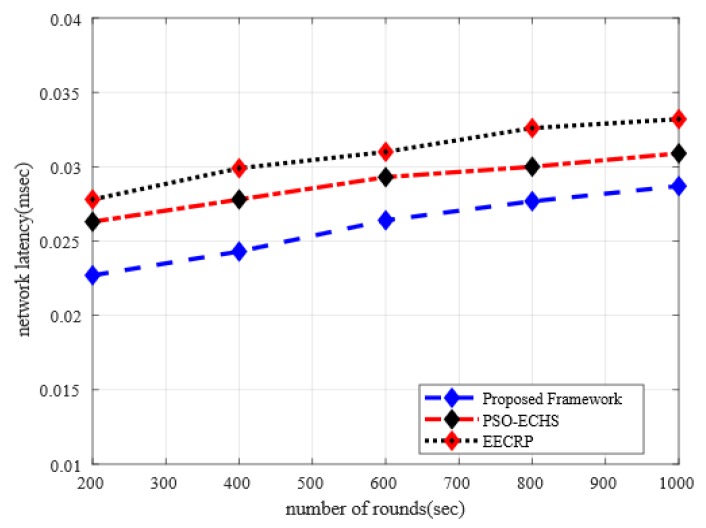
The impact of the simulation rounds on network latency.

Figure 5 illustrates the performance between the proposed framework and existing work concerning network latency. It increases the chances of network dis-connectivity and gives rise to a network latency ratio due to the presence of malicious nodes. However, it is observed from the experimental results that the network latency ratio of the proposed framework is improved by 11% and 17%, respectively, as compared to the existing solutions. The proposed framework chooses the most link and energy-efficient cluster heads for data routing towards BS. Moreover, the proposed framework exploits the SNR factor to identify the ratio of the congested link and reduces the chances of selecting the weak node as a data forwarder. Unlike a multi-hop paradigm that has the chances of a lot of obstacles on each hop, our proposed framework uses single-hop data transmission mode. Such an obstacle not only increases the network latency from cluster heads to BS, but on the other hand, it also reduces the energy consumption, when the transmission power of sensor nodes is considered in the measurement of network performance. Such a dynamic decision offers a longer lifetime routing path and agricultural data arrives at BS in a timely and efficient manner. Additionally, the proposed framework exploits the direct transmission path and decreases the ratio of network delay instead of using the multi-hop path, which significantly increases the ratio of network latency due to store and forward mechanism. Moreover, unlike most of the existing solutions that do not consider data security and lead to drop the data packet by the malicious node. Our proposed framework presents a secure algorithm for data encryption, which helps to avoid the data re-transmission and explicitly reduce the ratio of network delay between agricultural sensors and BS.

### 5.4. Analysis of Energy Consumption with Discussion

**Figure 6:**
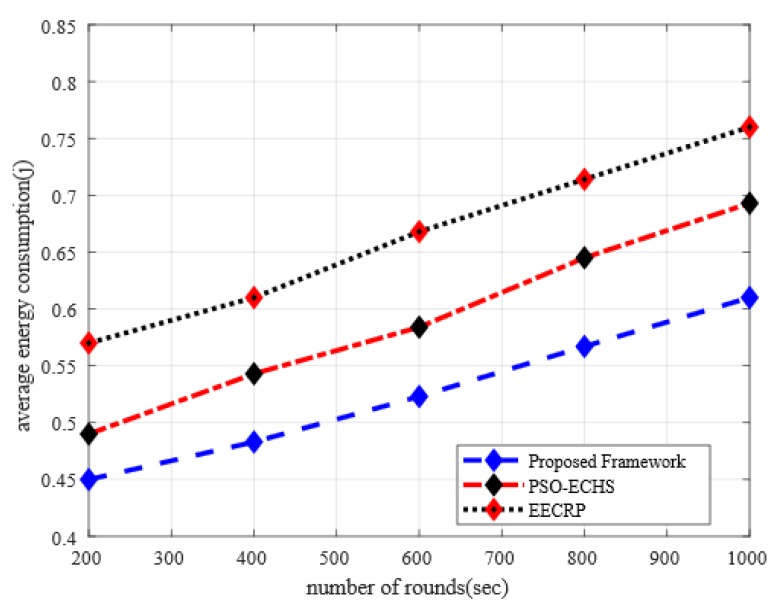
The impact of the simulation rounds on energy consumption.

In Figure 6, the performance analysis of the proposed framework in comparison of other solutions under varying simulation rounds is illustrated. It is seen from the simulation results that the proposed framework improves the ratio of energy consumption with other solutions by 11% and 21%. This is due to the proposed framework evenly distributing the load of energy consumption between sensor nodes. In the proposed framework, based on multi-criteria decision function an optimal nodes are selected for the position of clusters in agriculture land. Furthermore, the process of cluster heads selection and cluster formations are controlled by BS instead of in a distributed manner, which pointedly decreases the ratio of energy depletion in the observing field. Moreover, the process of recurrent re-clustering and re-routing are avoided by the proposed framework due to the multi-criteria decision function. The proposed framework significantly reduces the energy consumption in the agriculture domain, because the more appropriate cluster heads are selected in terms of energy efficiency, distance to BS, and signal strength factors.

### 5.5. Analysis of Routing Overheads with Discussion

**Figure 7:**
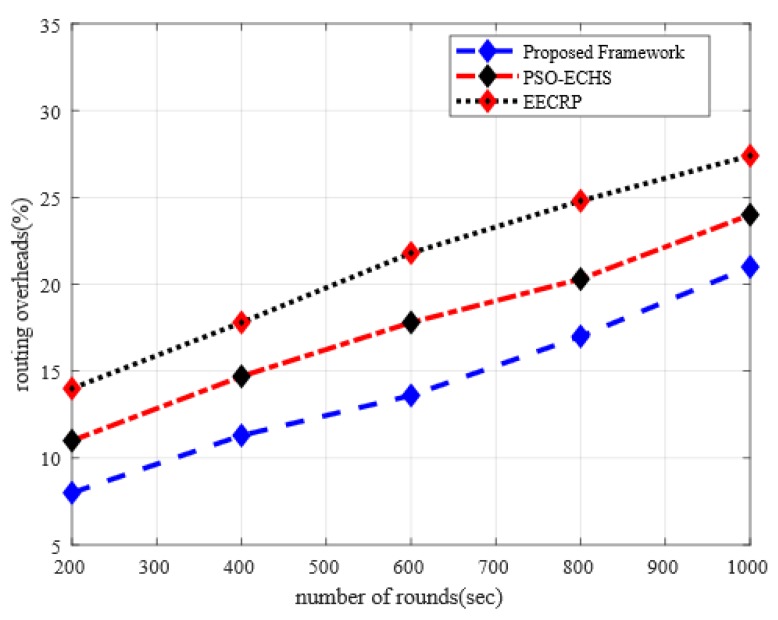
The impact of simulation rounds on routing overheads.

In Figure 7, the routing overheads between the proposed framework and other solution is illustrated under varying simulation rounds. The experimental results demonstrate that the proposed framework improves the routing overheads in comparison with the existing solutions by 19% and 33%, respectively. The proposed framework offers an intelligent decision that is based on multiple criteria for the selection of cluster heads with minimum processing overheads on sensor nodes. Besides, instead of using a multi-hop paradigm, the proposed framework exploits a single-hop pattern for data routing and makes use of distance to BS factor along with other performance parameters in routing decision. Such an approach decreases the probabilities of unnecessary energy consumption and overheads in data transmission. Additionally, unlike other solutions that maintain a local routing table on each node level and all the nodes update it frequently. The proposed framework maintains a global information table on BS and the table is updated in a controlled manner when needed. This mechanism expressively decreases the ratio of routing overheads and improves the performance of data transmission in the agriculture field. Moreover, the proposed framework drops the routing overheads under anonymous nodes by decreasing the possibility of data re-routing due to the integration of a data encryption algorithm based on the recurrence of the linear congruential generator.

## 6. Conclusions

The technology of wireless sensor networks performs a vital role in the development of the agriculture domain. This paper presents an energy-efficient and secure IoT based WSN framework for smart agriculture application. The main aim of the proposed framework is to appoint the more suitable cluster heads based on multi-criteria decision function. The decision is based on residual energy, distance to BS, and SNR factors. Additionally, the proposed framework is to adopt a single-hop paradigm for data transmission and decreases the chances of bottlenecks among agriculture sensors and BS. Our proposed framework presents an intelligent decision for data routing and decreases the ratio of energy consumption with improved data delivery performance in the agriculture field. Unlike most of the existing solutions, the proposed framework exploits a mechanism that is based on the SNR factor to determine the strength of signals and it achieves more stable network performance between agriculture sensors and BS. Moreover, the proposed framework offers secure data transmission from agriculture sensors towards BS based on secret keys while using the recurrence of the linear congruential generator. In future work, we aim to analyze the performance of the proposed framework in a mobile-based IoT network and Intelligent Transportation System (ITS).

## Figures and Tables

**Table 1:** Simulation parameters.

Parameter	Value
Simulation area	200 m × 200 m
Deployment	Random
Sensor nodes	100
Malicious nodes	15
Packet size, k	64 bits
Energy level	2 j to 4 j
Payload size	256 bytes
MAC layer	IEEE 802.11b
Control message	25 bits
Transmission range	20 m
Simulation rounds	0 to 1000
Traffic flows	CBR
Simulation tool	NS2.35

## References

[1] Dvir A., Ta V.T., Erlich S., Buttyan L. (2018). STWSN: A novel secure distributed transport protocol for wireless sensor networks. Int. J. Commun. Syst..

[2] Mehra P.S., Doja M.N., Alam B. (2018). Fuzzy based enhanced cluster head selection (FBECS) for WSN. J. King Saud Univ.-Sci..

[3] Tripathi A., Gupta H.P., Dutta T., Mishra R., Shukla K.K., Jit S. (2018). Coverage and connectivity in WSNs: A survey, research issues and challenges. IEEE Access.

[4] Shahzad M.K., Cho T.H. (2017). An energy-aware routing and filtering node (ERF) selection in CCEF to extend network lifetime in WSN. IETE J. Res..

[5] Zhang D.G., Zheng K., Zhang T., Wang X. (2015). A novel multicast routing method with minimum transmission for WSN of cloud computing service. Soft Comput..

[6] Awan K.A., Din I.U., Almogren A., Guizani M., Khan S. (2020). StabTrust—A Stable and Centralized Trust-Based Clustering Mechanism for IoT Enabled Vehicular Ad-Hoc Networks. IEEE Access.

[7] Din I.U., Guizani M., Kim B.S., Hassan S., Khan M.K. (2018). Trust management techniques for the Internet of Things: A survey. IEEE Access.

[8] Hamzah A., Shurman M., Al-Jarrah O., Taqieddin E. (2019). Energy-Efficient Fuzzy-Logic-Based Clustering Technique for Hierarchical Routing Protocols in Wireless Sensor Networks. Sensors.

[9] Kang S.H. (2019). Energy Optimization in Cluster-Based Routing Protocols for Large-Area Wireless Sensor Networks. Symmetry.

[10] Elshrkawey M., Elsherif S.M., Wahed M.E. (2018). An enhancement approach for reducing the energy consumption in wireless sensor networks. J. King Saud Univ.-Comput. Inf. Sci..

[11] Awan K.A., Din I.U., Zareei M., Talha M., Guizani M., Jadoon S.U. (2019). Holitrust-a holistic cross-domain trust management mechanism for service-centric Internet of Things. IEEE Access.

[12] Abuarqoub A., Hammoudeh M., Adebisi B., Jabbar S., Bounceur A., Al-Bashar H. (2017). Dynamic clustering and management of mobile wireless sensor networks. Comput. Netw..

[13] Lin C.C., Tseng P.T., Wu T.Y., Deng D.J. (2016). Social-aware dynamic router node placement in wireless mesh networks. Wirel. Netw..

[14] Khattak H.A., Ameer Z., Din U.I., Khan M.K. (2019). Cross-layer design and optimization techniques in wireless multimedia sensor networks for smart cities. Comput. Sci. Inf. Syst..

[15] Din I.U., Guizani M., Hassan S., Kim B.S., Khan M.K., Atiquzzaman M., Ahmed S.H. (2018). The Internet of Things: A review of enabled technologies and future challenges. IEEE Access.

[16] Awan K.A., Din I.U., Almogren A., Guizani M., Altameem A., Jadoon S.U. (2019). Robust trust–a pro-privacy robust distributed trust management mechanism for internet of things. IEEE Access.

[17] Din I.U., Guizani M., Rodrigues J.J., Hassan S., Korotaev V.V. (2019). Machine learning in the Internet of Things: Designed techniques for smart cities. Future Gener. Comput. Syst..

[18] Haseeb K., Almogren A., Islam N., Ud Din I., Jan Z. (2019). An Energy-Efficient and Secure Routing Protocol for Intrusion Avoidance in IoT-Based WSN. Energies.

[19] Haseeb K., Islam N., Almogren A., Din I.U., Almajed H.N., Guizani N. (2019). Secret Sharing-Based Energy-Aware and Multi-Hop Routing Protocol for IoT Based WSNs. IEEE Access.

[20] Hannah L., Donatti C.I., Harvey C.A., Alfaro E., Rodriguez D.A., Bouroncle C., Castellanos E., Diaz F., Fung E., Hidalgo H.G. (2017). Regional modeling of climate change impacts on smallholder agriculture and ecosystems in Central America. Clim. Chang..

[21] Zhou G., Zhou X., He Y., Shao J., Hu Z., Liu R., Zhou H., Hosseinibai S. (2017). Grazing intensity significantly affects belowground carbon and nitrogen cycling in grassland ecosystems: A meta-analysis. Glob. Chang. Biol..

[22] Abbasi A.Z., Islam N., Shaikh Z.A. (2014). A review of wireless sensors and networks’ applications in agriculture. Comput. Stand. Interfaces..

[23] Jawad H.M., Nordin R., Gharghan S.K., Jawad A.M., Ismail M. (2017). Energy-efficient wireless sensor networks for precision agriculture: A review. Sensors.

[24] Shinghal D., Srivastava N. (2010). Wireless Sensor Networks in Agriculture: For Potato Farming. Int. J. Eng. Sci..

[25] Alaparthy V.T., Morgera S.D. (2018). Multi-level intrusion detection system for wireless sensor networks based on immune theory. IEEE Access.

[26] Rawat P., Singh K.D., Chaouchi H., Bonnin J.M. (2014). Wireless sensor networks: A survey on recent developments and potential synergies. J. Supercomput..

[27] Balamurali R., Kathiravan K. (2015). An analysis of various routing protocols for Precision Agriculture using Wireless Sensor Network. Proceedings of the 2015 IEEE Technological Innovation in ICT for Agriculture and Rural Development (TIAR).

[28] Banđur Đ., Jakšić B., Banđur M., Jović S. (2019). An analysis of energy efficiency in Wireless Sensor Networks (WSNs) applied in smart agriculture. Comput. Electron. Agric..

[29] Zia H., Harris N.R., Merrett G.V., Rivers M., Coles N. (2013). The impact of agricultural activities on water quality: A case for collaborative catchment-scale management using integrated wireless sensor networks. Comput. Electron. Agric..

[30] Yu Y., Liu J. (2018). An Energy-Aware Routing Protocol with Small Overhead for Wireless Sensor Networks. Proceedings of the International Conference on Data Mining and Big Data.

[31] Ullah U., Khan A., Zareei M., Ali I., Khattak H.A., Din I.U. (2019). Energy-effective cooperative and reliable delivery routing protocols for underwater wireless sensor networks. Energies.

[32] Haseeb K., Islam N., Almogren A., Din I.U. (2019). Intrusion Prevention Framework for Secure Routing in WSN-Based Mobile Internet of Things. IEEE Access.

[33] Darabkh K.A., Albtoush W.Y., Jafar I.F. (2017). Improved clustering algorithms for target tracking in wireless sensor networks. J. Supercomput..

[34] Enam R.N., Qureshi R., Misbahuddin S. (2014). A uniform clustering mechanism for wireless sensor networks. Int. J. Distrib. Sens. Netw..

[35] Zhu C., Wu S., Han G., Shu L., Wu H. (2015). A tree-cluster-based data-gathering algorithm for industrial WSNs with a mobile sink. IEEE Access.

[36] Heinzelman W.R., Chandrakasan A., Balakrishnan H. (2000). Energy-efficient communication protocol for wireless microsensor networks. in System Sciences, 2000. Proceedings of the 33rd Annual Hawaii International Conference.

[37] Karaca O., Sokullu R., Prasad N.R., Prasad R. (2012). Application oriented multi criteria optimization in WSNs using on AHP. Wirel. Pers. Commun..

[38] Jain B., Brar G., Malhotra J. (2018). EKMT-k-means clustering algorithmic solution for low energy consumption for wireless sensor networks based on minimum mean distance from base station. Networking Communication and Data Knowledge Engineering.

[39] Azad P., Sharma V. (2013). Cluster head selection in wireless sensor networks under fuzzy environment. ISRN Sens. Netw..

[40] Lung C.H., Zhou C. (2010). Using hierarchical agglomerative clustering in wireless sensor networks: An energy-efficient and flexible approach. Ad. Hoc. Netw..

[41] Wu H., Zhu H., Zhang L., Song Y. (2019). Energy Efficient Chain Based Routing Protocol for Orchard Wireless Sensor Network. J. Electr. Eng. Technol..

[42] Srivastava J.R., Sudarshan T.S.B. (2015). A genetic fuzzy system based optimized zone based energy efficient routing protocol for mobile sensor networks (OZEEP). Appl. Soft Comput..

[43] Rao P.S., Jana P.K., Banka H. (2017). A particle swarm optimization based energy efficient cluster head selection algorithm for wireless sensor networks. Wirel. Netw..

[44] Shen J., Wang A., Wang C., Hung P.C., Lai C.F. (2017). An efficient centroid-based routing protocol for energy management in WSN-assisted IoT. IEEE Access.

[45] Ali R., Pal A.K., Kumari S., Karuppiah M., Conti M. (2018). A secure user authentication and key-agreement scheme using wireless sensor networks for agriculture monitoring. Future Gener. Comput. Syst..

[46] Ripley B.D. (1990). Thoughts on pseudorandom number generators. J. Comput. Appl. Math..

[47] L’Ecuyer P., Andres T.H. (1997). A random number generator based on the combination of four LCGs. Math. Comput. Simul..

[48] Goyal M., Prakash S., Xie W., Bashir Y., Hosseini H., Durresi A. Evaluating the impact of signal to noise ratio on IEEE 802.15. 4 PHY-level packet loss rate. Proceedings of the 2010 13th International Conference on Network-Based Information Systems.

[49] Lavrador P.M., de Carvalho N.B., Pedro J.C. (2004). Evaluation of signal-to-noise and distortion ratio degradation in nonlinear systems. IEEE Trans. Microw. Theory Tech..

[50] Ali M.S., Dey T., Biswas R. (2008). ALEACH: Advanced LEACH routing protocol for wireless microsensor networks. Proceedings of the 2008 International Conference on Electrical and Computer Engineering.

[51] Tripathi M., Gaur M.S., Laxmi V. Simulation of Snooze attack in LEACH. Proceedings of the 3rd International Conference of Computer Science, Engineering and Applications (ICCSEA’13).

[52] Wang A., Yang D., Sun D. (2012). A clustering algorithm based on energy information and cluster heads expectation for wireless sensor networks. Comput. Electr. Eng..

[53] Ibrihich O., Krit S.D., Laassiri J., El Hajji S. (2015). Study and Simulation of Protocols of WSN Using NS2. Transactions on Engineering Technologies.

[54] Issariyakul T., Hossain E. (2009). An Introduction to Network Simulator NS2.

